# Persistent cognitive deficits in ACL-injured athletes despite of rehabilitation: an observational longitudinal study

**DOI:** 10.3389/fspor.2025.1601744

**Published:** 2025-07-15

**Authors:** Jesús Jiménez-Martínez, Francisco Alarcón-López, Luis Javier Chirosa-Ríos, Alejandro Gutiérrez-Capote, David Cárdenas-Vélez

**Affiliations:** ^1^ Department of Physical Education and Sport, Faculty of Sports Science, University of Granada, Granada, Spain; ^2^ Department of Physical Education and Sports, Sport and Health University Research Institute (iMUDS), University of Granada, Granada, Spain; ^3^ Department of General and Specific Didactics, Faculty of Education, University of Alicante, Alicante, Spain

**Keywords:** ACL injury, cognitive inhibition, rehabilitation, cognitive deficit, executive functions

## Abstract

**Background:**

Rehabilitation programs following an anterior cruciate ligament (ACL) injury tend to focus on improving conditional aspects such as biomechanics. Recently, some studies have analyzed the relationship between cognition and ACL injury, but how cognitive performance evolves throughout the rehabilitation process has not yet been explored. This study assessed how cognitive performance evolves at three points in the ACL injury recovery process: preoperatively, postoperatively, and at the end of the rehabilitation process. It also aims to compare cognitive performance at the end of the rehabilitation process with athletes without a history of ACL injury.

**Methods:**

30 open-skill sports athletes who had recently sustained an ACL injury and 30 open-skill sports athletes with no history of ACL injury were recruited. For the group of ACL-injured athletes, three experimental sessions were conducted at three different points in the ACL injury recovery process. For the control group a single experimental session was conducted. During the experimental sessions participants performed Flanker Task and Multiple Object Tracking to evaluate their cognitive performance.

**Results:**

For both Flanker and MOT task, ACL injury athletes show better cognitive performance postoperatively compared to the preoperative phase. For example, a higher mean reaction time in the Flanker task (BF₁₀ = 4.14) and lower accuracy in 3-ball tracking at 28.8 deg/s (BF₁₀ = 2.45). Nevertheless, no improvement was observed between the postoperative and follow-up phases. Finally, ACL injury athletes did not reach a cognitive performance comparable to healthy athletes, for example on mean reaction time in the Flanker Task (BF₁₀ = 60.64) and the 3-ball tracking at speeds of 19.9 and 28.8 deg/s (BF₁₀ = 16.30, BF₁₀ = 12.12, respectively).

**Conclusions:**

ACL injury athletes show improvements in cognitive performance post-surgery, but it stabilizes at the end of the rehabilitation and remains lower than that of athletes who did not suffer an ACL injury. Therefore, ACL rehabilitation programs fail to improve cognitive performance, increasing the risk of suffering a new ACL injury compared to those without a history of ACL injury.

## 1 Introduction

The return to competition for athletes who have suffered an anterior cruciate ligament (ACL) injury does not consistently produce the expected outcomes. On the one hand, a 17% of these athletes do not return to competition. Indeed, just a 53% regain their pre-injury sporting level (1). On the other hand, these athletes face the risk of recurrence, as it has been observed that up to 23% of athletes who return to activity suffer a new ACL injury (2). This circumstance is more common in open-skill sports where the physical and mental demands are particularly high (3). According to data from a cohort of 3,482 internal knee injuries (4), ACL injuries were markedly more common in interaction sports such as soccer or ski (82.5%), while individual and non-open skills sports combined represented only 17.5%.

Rehabilitation programs following an ACL injury tend to focus on improving conditional aspects such as strength and joint range of motion, which aligns with the aims of many studies investigating the predisposing factors for ACL injury (5). Furthermore, these programs are characterized by closed motor skill work, mainly through predictable and controlled tasks that have limited transfer to the demands of open-skill sports (6). In the competitive context of this type of sport, actions are dynamic and unpredictable and require quick decisions in response to external stimuli, such as opponents' movements or the trajectory of the ball.

In open-skill sports, the high cognitive demands of the game are associated with mechanisms linked to ACL injury risk, such as axial compression forces or knee valgus, which lower the tolerance threshold of ligamentous tissue (7). These mechanisms are often activated without physical contact, especially in abrupt direction changes, decelerations, or single-leg landings (8). In these contexts, the ACL has difficulty preventing anterior tibial translation, rotational movements, and knee hyperextension (9). In fact, between 72% and 95% of ACL injuries occur during dynamic movements, such as direction changes, without physical contact (10).

Cognitive abilities play a crucial role in reducing the risk of injury in open-skill sports, where athletes must continuously adapt to unpredictable and rapidly changing environments. Elite athletes demonstrate superior performance in basic executive function tasks (11–13), as well as in other attentional control paradigms, which highlights that efficient executive functioning allows athletes to filter irrelevant information, make rapid decisions, and execute appropriate motor responses in high-pressure contexts effectively (14). Conversely, deficits in these cognitive domains have been associated with a higher risk of musculoskeletal injuries, particularly during tasks that require quick adjustments, such as landings or directional changes (15, 16). For example, lower performance in tasks involving attentional tracking (e.g., Multiple Object Tracking, MOT) and motor inhibition has been linked to an increased risk of injury, especially in sports characterized by high cognitive and physical demands (17). In this context, athletes with stronger cognitive abilities may be better equipped to anticipate and respond to external stimuli, thereby mitigating the biomechanical risks associated with non-contact ACL injuries (18–20).

There also appears to be a relationship between the level of performance in these cognitive skills and a lower risk of musculoskeletal injury (16). Along the same lines, it has been observed that in noncontact ACL injuries, there is an impairment in inhibitory and attentional control during the execution of motor actions in football players (17). This impairment may be because the ligament rupture generates changes at the neural level due to altered afferent signaling (21). This alteration affects the information transmitted by mechanoreceptors to the central nervous system, which can have detrimental consequences in the somatosensory and motor areas of the brain (22). As a result, brain reorganization may require compensatory strategies, which may not be sufficient in competitive and dynamic contexts to make the necessary motor adjustments, thus increasing the risk of injury (23). However, it is common to observe a lack of strategies oriented to the control and readjustment of cognitive status in ACL injury prevention, rehabilitation, and readaptation programs, which increases the risk of recurrence.

Although there are studies that have analyzed the relationship between cognition and ACL injury, how cognitive performance evolves throughout the rehabilitation process has not yet been explored (24). Consequently, the present study aims to analyze how cognitive performance evolves at three points in the ACL injury recovery process: preoperatively, postoperatively, and at the end of the recovery/rehabilitation process. It also aims to compare cognitive performance at the end of the recovery/rehabilitation process with athletes without a history of ACL injury. It was hypothesised that the ACL injury recovery programs would not be sufficient to restore athletes' cognitive performance fully, and that athletes without a history of ACL injury would demonstrate superior cognitive performance compared to previously injured athletes at follow-up. This study will provide relevant information to rehabilitators and physical therapists on how cognitive performance evolves after injury to improve their intervention programs and avoid the risk of recurrence.

## 2 Materials and methods

### 2.1 Ethical approval

The study was conducted in accordance with the Declaration of Helsinki and was approved by the university institutional ethics committee (approval number: 3110/CEIH/2022).

### 2.2 Participants

A total of 30 open-skill sports athletes who had recently sustained an ACL injury were recruited. In addition, 30 open-skill sports athletes with no history of ACL injury were recruited. Data were collected on age, weight, height, and the sports discipline practiced by each participant. Furtheremore, participants in the ACL-injured group were recruited through different physiotherapy clinics and digital platforms, and therefore, the rehabilitation protocols they followed may have varied and were not standardized across the sample. Table 1 presents a summary of the demographic characteristics of the participants.

**Table 1:** Participant's demographic data.

Demographic data	ACLR athletes	Healthy athletes
*N* (Male/Female)	20/10	19/11
Years of practical experience	8.17 ± 1.86	7.98 ± 1.24
Age (years)	24.14 ± 5.33	22.29 ± 4.41
Height (m)	1.75 ± 0.22	1.77 ± 0.26
Body mass (kg)	77.23 ± 9.56	78.21 ± 9.34
Sport modality	Football (17) Basketball (8) Handball (3) Volleyball (2)	Matched to ACLR Athletes group
Time between ACL tear and first testing (days)	7.65 ± 2.48	Not applicable
Time between ACLR and second testing (months)	2.19 ± 0.36	Not applicable
Time between ACLR and final testing (years)	1.27 ± 0.53	Not applicable

Data values are expressed as mean ± standard deviation. *N*, sample size; M, meters; Kg, kilograms; ACL, anterior cruciate ligament; ACLR, anterior cruciate ligament reconstruction.

To be included in the study, participants had to meet the following selection criteria: (1) be young adults between 18 and 35 years old, (2) not have a diagnosis of psychiatric or neurological disorders (such as ADHD, depression, among others), (3) not have a history of brain injury or severe head trauma, (4) not have been diagnosed with cardiovascular and/or metabolic diseases, (5) have normal or corrected vision, and (6) be active athletes in open-skill sports, with a minimum of two discipline-specific training sessions per week and at least three years of continuous experience in federated sports competitions, (7) have suffered a total ACL rupture during the two weeks prior to the first experimental session. Finally, the control group of healthy athletes had no ACL injury history and met the other selection criteria previously indicated.

The sample size was determined concerning previous studies employing similar study designs, ensuring methodological consistency and adequate statistical power (25).

### 2.3 Procedure

The present study had two experimental groups. The first group consisted of ACL-injured athletes whose cognitive performance was monitored throughout recovery. The second group consisted of athletes with no history of ACL injury who acted as a control group. This allowed a comparison of cognitive performance at the end of the recovery phase with cognitive performance in athletes with no history of ACL injury.

#### 2.3.1 Familiarization session

In the familiarisation session, participants received a detailed presentation of the research and detailed instructions on the procedures and assessments they would carry out in the experimental sessions. In addition, participants gave their written consent to participate. They were then able to practice all the cognitive tests until they had a full understanding of them. This session lasted approximately 60 min and was held one to two days prior to the first experimental session. Finally, this familiarisation session was conducted in the laboratory at the Faculty of Physical Activity and Sport Sciences of the University of Granada, and which took place post-injury but preoperatively, within the first 15 days following ACL rupture.

#### 2.3.2 Experimental session

For the group of ACL-injured athletes, three experimental sessions were conducted at three different points in the ACL injury recovery process. The athletes were evaluated within the first 15 days after the total rupture of the ACL, specifically 7.65 ± 2.48 days post-injury, during the preoperative phase. In rehabilitation, athletes were evaluated during the four months following ligament reconstruction, approximately 2.19 ± 0.36 months after surgical reconstruction. Finally, the last experimental session was conducted to evaluate the athletes once the rehabilitation process was completed (they had to be medically cleared to return to competition) on average 1.27 ± 0.53 years after surgery. In the case of the control group, i.e., the athletes with no history of ACL injury, a single experimental session was conducted to assess their cognitive performance.

Comparisons were made to examine the evolution of cognitive performance throughout recovery. To do so, intra-group comparisons were performed across the three assessment phases for the ACL-injured athletes' group. Additionally, inter-group comparisons were conducted between the ACL-injured athletes (at the end of the recovery phase) and the healthy control group. The end of the recovery phase also referred to as the follow-up assessment, was defined as the moment when athletes had been medically cleared to return to unrestricted sports participation.

The experimental sessions were conducted in a quiet and undisturbed laboratory at the Faculty of Physical Activity and Sport Sciences of the University of Granada. To control for possible moderating variables of cognitive performance, participants had to fulfill certain requirements before the experimental session: abstain from caffeine or theine 12 h before, not drink alcohol 24 h before, fast for the previous 4 h, not perform strenuous exercise during the previous 48 h, and sleep at least 7 h the night before. The session lasted approximately 45 min. Although the time of day was not strictly controlled, all testing sessions were conducted between 9:00 a.m. and 6:00 p.m., and each participant was assessed at approximately the same time of day across sessions whenever possible to reduce variability related to circadian influences. Finally, at the beginning of each experimental session, participants completed a brief familiarization trial for each cognitive task to ensure they recalled the instructions and understood the task demands before formal testing began.

Participants completed two computer-based cognitive tasks, and the order of task administration was randomized for each participant at baseline. Task order was randomized using a simple AB/BA counterbalancing procedure. Half of the participants were randomly assigned to complete the Flanker Task first followed by the MOT task (AB), while the other half completed the tasks in the reverse order (BA). This random assignment determined whether the Flanker Task or the MOT task was performed first. The assigned task order was maintained consistently across all three assessment time points throughout the recovery process to control for order effects. A 10-minute rest period was provided between the two cognitive tasks to minimise potential fatigue and carry-over effects. Participants also received immediate feedback on their performance during the execution of the cognitive tasks, which helped maintain task engagement and ensured understanding of response accuracy. These tests assessed basic executive functions such as cognitive inhibition (interference control). These variables have been previously studied in athletes with a history of ACL injury (26).

### 2.4 Variables and instruments

#### 2.4.1 Flanker task

This task aimed to assess interference control (27). Participants were instructed to respond to the direction of a central white arrow displayed on a computer screen. They had to press the left shift key with their left index finger if the central arrow pointed to the left (‘<’), and the right shift key with their right index finger if the central arrow pointed to the right (‘>’). Congruent trials consisted of flanking arrows pointing in the same direction as the central arrow, while incongruent trials had flankers pointing in the opposite direction. The task included four different conditions:

1. Congruent: All arrows pointed in the same direction (e.g., ‘< < < < < **<** < < < < <’ or ‘> > > > > **>** > > > > >’).
2. Incongruent: The central arrow pointed in the opposite direction to the surrounding arrows (e.g., ‘> > > > > **<** > > > > >’ or ‘< < < < < **>** < < < < <’).
3. Neutral: A central arrow with no surrounding arrows (e.g., ‘<’ or ‘>’).
4. Dash: A central arrow flanked by neutral dashes (e.g., ‘–<’ or ‘–>’).

The task consisted of 172 trials divided into blocks: 12 practice trials, followed by 40 trials for each condition (congruent, incongruent, neutral, and dashes), presented in random order. Each trial began with a fixation cross displayed for 500 ms, followed by an arrow stimulus for 800 ms. A 1,000 ms interval was included between trials. The main measures of interest were accuracy and mean response time in each condition.

The Flanker Task was administered using the Psychology Experiment Building Language (PEBL) software, version 2.0 (28).

#### 2.4.2 Multiple object tracking

**Figure 1:**
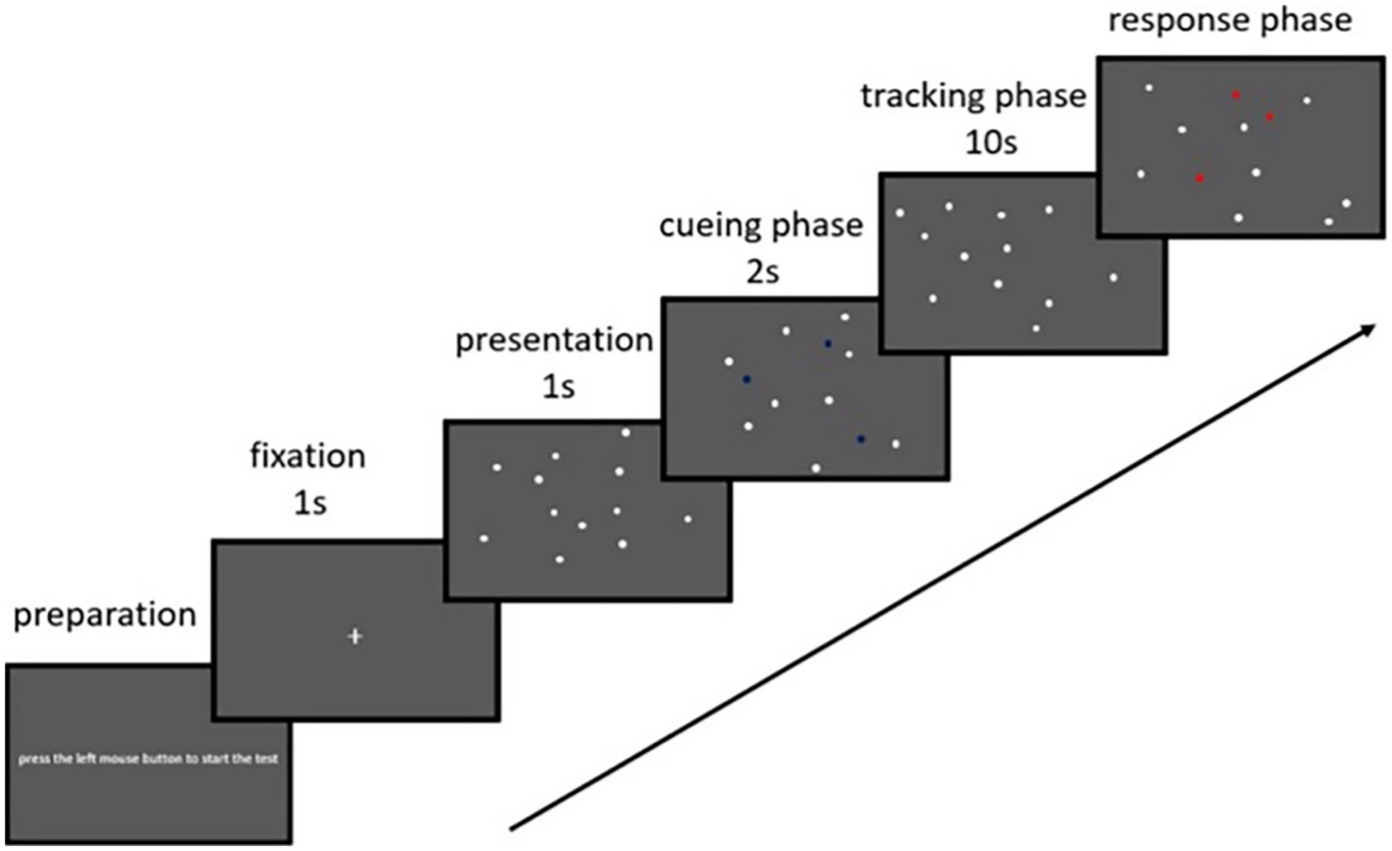
MOT task representation. Extracted from (50).

The multiple object tracking (MOT) task is a widely used method to investigate working memory (29) and dynamic continuous attention (30), especially in skills such as maintaining sustained attention, distributing attention among multiple objects, and selecting specific items for active tracking. The MOT task is graphically represented in Figure 1.

In this test, eight identical, white-colored spheres (radius = 0.4°) were presented were presented against a gray background on a 27-inch monitor placed 50 cm away from the participant at a visual angle 25°. At the beginning of each trial, a white fixation cross appeared for 1,000 ms to center attention. For 2 s, two or three of the spheres (depending on the condition) were randomly illuminated green to mark them as targets. Subsequently, all spheres returned to white and began to move in randomly generated trajectories across the screen at one of five predefined speeds (6.4°/s, 9.3°/s, 13.7°/s, 19.9°/s, and 28.8°/s). Sphere motion was updated at 60 Hz and constrained by screen borders and collision rules. The participant was required to mentally track the initially illuminated spheres for 5 s mentally. After stopping, each sphere was randomly assigned a number from 1 to 8, and the participant had to identify the originally illuminated targets by indicating their corresponding numbers.

The spheres' movement speed varied in five levels (6.4 deg/s, 9.3 deg/s, 13.7 deg/s, 19.9 deg/s, and 28.8 deg/s), with five repetitions for each speed. Then, the same procedure was applied, but with three spheres to follow. During the test, no instructions were given that could influence the participant's performance.

Performance was determined by calculating the average percentage of hits for each speed. For example, when tracking three spheres at a speed of 28.8 deg/s, the total hits were summed and divided by 0.15, corresponding to the maximum possible 15 hits.

The MOT task was programmed and executed using PsychoPy (version 2023.1.2).

### 2.5 Statistical analysis

All analyses were performed using JASP 0.19.3. The main objective of the statistical analysis was to interpret the null results, so a Bayesian approach was chosen. In Bayesian inference, the Bayes Factor (BF) is a measure expressed as a ratio that reflects the degree of support the observed data provide for two competing models: one corresponding to the null hypothesis and the other to the alternative hypothesis. The BF allows updating the relative belief in one hypothesis vs. the other, considering certain prior assumptions (31). Depending on the value of the BF, three possible interpretations can be obtained: evidence in favor of the alternative hypothesis model, evidence in favor of the null hypothesis model, or inconclusive results. Although a larger sample size usually increases the likelihood of obtaining strong evidence (either in favor of the null or alternative hypothesis), Bayes Factors can be interpreted independently of sample size. Nevertheless, although Bayes Factors are generally less sensitive to sample size than frequentist *p*-values, they are not entirely independent of it, particularly when the evidence is classified as anecdotal or inconclusive. Since the preoperative measure had a smaller sample size than the other measures, this type of analysis is the most appropriate for interpreting the data in this study. It is important to highlight that the preoperative sample size was smaller due to the logistical difficulty of recruiting athletes during the short window between injury and surgical intervention.

All repeated measures ANOVA analyses were conducted using the default JASP settings. This setting includes an r-scale value 0.5 for fixed-effect priors to avoid *a priori* mass dispersion at excessively large effect sizes (32). Where necessary, alternative values of 0.2 and 1 were also used to ensure the results' robustness.

The ACL injury recovery phase was predicted to be ineffective from a cognitive perspective. Therefore, evidence was sought to support the null hypothesis (H0) over the alternative directional hypothesis (H1), which suggested that the postoperative measure would be superior to the preoperative phase and that the follow-up measure would be superior to both. In implementing Bayes Factors (BF) used in this study, the null model is represented by a point null hypothesis (where the effect of interest is zero, implying that the ACL recovery phase is ineffective in restoring cognitive abilities). Instead, the alternative model is a composite hypothesis, where the median of the expected effect and its standard deviation are determined by selecting a prior distribution (a zero-truncated Cauchy distribution with a width of 0.707 as default), and using Bayesian repeated-measures ANOVA tests with *post hoc* analysis in JASP to assess the possible superiority of the follow-up phase over the postoperative phase, and the latter over the preoperative phase. Following the most common qualitative interpretation (33), substantial support for the null hypothesis exists when the Bayes Factor (BF_10_) is less than 0.33.

As a secondary prediction, it was established that athletes without a history of ACL injury would demonstrate superior cognitive performance on the follow-up measures. To test this hypothesis, a Bayesian Independent Samples T-Test was conducted on the Flanker and MOT tasks, considering the superiority of uninjured athletes as the alternative hypothesis. In this case, substantial support for the alternative hypothesis would be reflected in a Bayes Factor (BF_10_) greater than 3.

## 3 Results

### 3.1 Evolution of inhibitory control throughout the three phases of the ACL recovery process

#### 3.1.1 Mean RT

Fot the overall mean reaction time, the repeated measures ANOVA analysis, followed by *post hoc* comparisons between the preoperative and postoperative phases yielded a Bayes factor BF₁₀ = 4.14, providing evidence in favor of the alternative hypothesis, suggesting an effect of the retrieval process. Similarly, the *post hoc* comparisons between the preoperative and follow-up phases showed a BF_10_ = 15.03, strongly supporting an effect. However, the *post hoc* comparisons between the postoperative phase and the follow-up phase obtained a BF₁₀ = 0.26, indicating substantial evidence in favor of the null hypothesis and, therefore, the absence of an additional effect between these stages.

#### 3.1.2 Congruent trials RT

For the reaction time in congruent trials, the repeated measures ANOVA, followed by *post hoc* comparisons between the preoperative and postoperative phases yielded a BF₁₀ = 5.66. In contrast, the comparison between the preoperative and follow-up phases resulted in a BF₁₀ = 15.14, in both cases providing substantial evidence in favor of the alternative hypothesis. In contrast, the *post hoc* comparisons between the postoperative and follow-up phases showed a BF₁₀ = 0.28, supporting the null hypothesis and indicating the absence of an additional effect.

#### 3.1.3 Incongruent trials RT

For reaction time on incongruent trials, the repeated measures ANOVA, followed by *post hoc* comparisons between the preoperative and postoperative phases obtained a BF₁₀ = 0.40, while that between the preoperative and follow-up phases resulted in a BF₁₀ = 0.53, in both cases suggesting inconclusive results. Furthermore, the *post hoc* comparisons between the postoperative phase and the follow-up phase showed a BF₁₀ = 0.27, which provides substantial evidence in favor of the null hypothesis, indicating the absence of effect.

#### 3.1.4 Number of errors

Regarding the number of errors, the repeated measures ANOVA, followed by *post hoc* comparisons between the preoperative and postoperative phases showed a BF₁₀ = 0.60, while that between the preoperative and follow-up phases resulted in a BF₁₀ = 0.59, in both cases providing inconclusive results. Meanwhile, the *post hoc* comparisons between the postoperative phase and the follow-up phase obtained a BF₁₀ = 0.29, which supports the null hypothesis and suggests the absence of an effect.

#### 3.1.5 Accuracy

For mean accuracy, the repeated measures ANOVA, followed by *post hoc* comparisons between the preoperative and postoperative phases showed a BF₁₀ = 0.47, while that between the preoperative and follow-up phases resulted in a BF₁₀ = 0.49, in both cases providing inconclusive results. Likewise, the *post hoc* comparisons between the postoperative and follow-up phases obtained a BF_10_ = 0.43, suggesting anecdotal evidence favoring the null hypothesis and, therefore, the absence of an effect.

#### 3.1.6 Switching cost

Finally, regarding the switching cost (the difference in reaction time between congruent and incongruent trials), the repeated measures ANOVA, followed by *post hoc* comparisons between the preoperative and postoperative phases showed a BF₁₀ = 0.42, suggesting inconclusive results. Similarly, the *post hoc* comparisons between the preoperative and follow-up phases obtained a BF₁₀ = 0.29, providing substantial evidence of no effect. Finally, the *post hoc* comparisons between the postoperative and follow-up phases yielded a BF₁₀ = 0.27, anecdotally supporting the null hypothesis and suggesting no further switching in this phase.

Finally, to ensure the robustness of the results, wider (*r* = 1) and narrower (*r* = 0.2) priors were evaluated for these effects. Overall, the interpretation of the results was not affected by variations in prior dispersion. For example, for the mean overall reaction time, BF_10_ = 129.77 was obtained with prior *r* = 0.05, BF_10_ = 67.85 with prior *r* = 0.02, and BF_10_ = 154.33 with prior *r* = 1, results that continue to indicate evidence in favor of the alternative hypothesis. The results regarding means (SD) and Bayes factors BF_10_ are reported in Table 2.

**Table 2:** Performance on the flanker task at the three moments of the recovery process.

Flanker task variables	Pre-surgery			Post-surgery			Follow-up					
	M (SD)	95% credible interval		M (SD)	95% credible interval		M (SD)	95% credible interval		Pre-surgery vs. post-surgery BF_10_	Pre-surgery vs. follow-up BF_10_	Post-surgery vs. follow-up BF_10_
		Lower	Upper		Lower	Upper		Lower	Upper			
Mean RT	412.98 (40.68)	387.14	438.83	390.65 (41.13)	364.52	416.79	385.17 (43.39)	357.60	412.73	4.14	15.03	0.26
Congruent RT	405.58 (41.13)	379.45	431.76	382.08 (37.86)	358.02	406.13	378.14 (41.04)	352.06	404.21	5.66	15.14	0.28
Incongruent RT	452.37 (41.38)	426.08	478.66	450.21 (31.78)	430.02	470.41	443.08 (32.77)	422.26	463.91	0.40	0.53	0.27
Errors	9.17 (4.22)	6.49	11.85	7.83 (4.71)	4.84	10.82	7.67 (5.43)	4.22	11.12	0.60	0.59	0.29
Mean accuracy	0.94 (0.03)	6.487	11.846	0.95 (0.03)	4.843	10.824	0.95 (0.03)	4.215	11.118	0.47	0.49	0.43
Cost of change	48.34 (13.72)	39.621	57.059	51.85 (7.31)	47.205	56.489	48.31 (8.46)	42.931	53.679	0.42	0.29	0.27

Data values are expressed as mean (standard deviation); ACLR, anterior cruciate ligament reconstruction; BF_10_, Bayes factor.

### 3.2 Evolution of cognitive performance in the MOT task throughout the three phases of the ACL recovery process

For the MOT test overall, BF₁₀ values for the other 2-ball conditions do not show consistent changes in performance, with values mostly falling within a range that anecdotally supports the null hypothesis. In the 3-ball, 6.4 deg/s condition, performance remained stable between phases (BF_10_ = 0.44 for all comparisons). For higher velocities, results show subtle improvements postoperatively, especially for the 3-ball 19.9 deg/s model, where an increase in accuracy was observed in the follow-up phase compared to the preoperative phase (BF_10_ = 3.25). Similarly, for the 3-ball 28.8 deg/s model, there was an increase in accuracy postoperatively with a BF_10_ = 2.45, suggesting anecdotal evidence in favor of the alternative hypothesis and an even more evident improvement in the follow-up phase (BF_10_ = 5.73), suggesting a progressive benefit in recovery. However, inconclusive results were observed when comparing the last two recovery phases. As in the Flanker task, the results were not affected by variations in prior dispersion for either the *r* = 0.02 or *r* = 1 prior. For example, for the 3-ball velocity at 28.8 deg/s, BF_10_ = 12.80 was obtained with the *r* = 0.05 prior, BF_10_ = 8.39 with the *r* = 0.02 prior, and BF_10_ = 11.44 with the *r* = 1 prior, results that continue to indicate evidence in favor of the alternative hypothesis. The results regarding means (SD) and Bayes factors BF_10_ are reported in Table 3.

**Table 3:** Performance on the MOT task at the three moments of the recovery process.

MOT task variables	Pre-surgery			Post-surgery			Follow-up					
	M (SD)	95% credible interval		M (SD)	95% credible interval		M (SD)	95% credible interval		Pre-surgery vs. post-surgery BF_10_	Pre-surgery vs. follow-up BF_10_	Post-surgery vs. follow-up BF_10_
		Lower	Upper		Lower	Upper		Lower	Upper			
2 balls (6.4 deg/s)	99.17 (2.89)	91.33	101.00	95.00 (5.22)	91.68	98.32	95.83 (6.69)	91.59	100.08	0.83	0.69	0.30
2 balls (9.3 deg/s)	95.00 (5.22)	91.68	98.32	95.83 (5.15)	92.56	99.11	96.67 (4.92)	93.54	99.80	0.33	0.44	0.31
2 balls (13.7 deg/s)	84.17 (15.64)	74.23	94.11	90.00 (15.95)	79.86	100.14	89.33 (13.11)	75.72	97.62	1.76	0.40	0.31
2 balls (19.9 deg/s)	70.00 (12.06)	62.34	77.66	66.67 (13.71)	57.96	75.38	74.17 (10.84)	67.28	81.05	0.39	0.50	1.85
2 balls (28.8 deg/s)	48.33 (16.97)	37.55	59.11	56.67 (17.75)	45.39	67.95	53.33 (17.75)	42.05	64.61	0.67	0.42	0.37
3 balls (6.4 deg/s)	99.44 (1.93)	98.21	100.67	98.89 (2.60)	97.23	100.54	98.88 (2.61)	97.23	100.54	0.44	0.44	0.44
3 balls (9.3 deg/s)	95.83 (6.84)	91.48	100.17	97.22 (6.00)	93.40	101.03	97.56 (6.00)	93.40	101.03	0.41	0.40	0.28
3 balls (13.7 deg/s)	83.66 (10.39)	75.06	88.27	85.55 (10.60)	78.83	92.26	86.33 (9.15)	82.51	94.15	0.95	1.30	0.65
3 balls (19.9 deg/s)	65.27 (9.99)	58.92	71.62	70.77 (13.87)	58.96	76.59	72.50 (11.55)	65.16	79.84	0.35	3.25	0.57
3 balls (28.8 deg/s)	40.82 (11.02)	33.82	47.82	49.99 (12.86)	41.82	58.16	51.94 (3.42)	44.41	59.47	2.45	5.73	0.68

Data values are expressed as mean (standard deviation). ACLR, anterior cruciate ligament reconstruction; deg, degrees; s, seconds; BF_10_, Bayes factor.

### 3.3 Comparison of cognitive performance between athletes without a history of ACL injury and athletes in the follow-up phase

**Figure 2:**
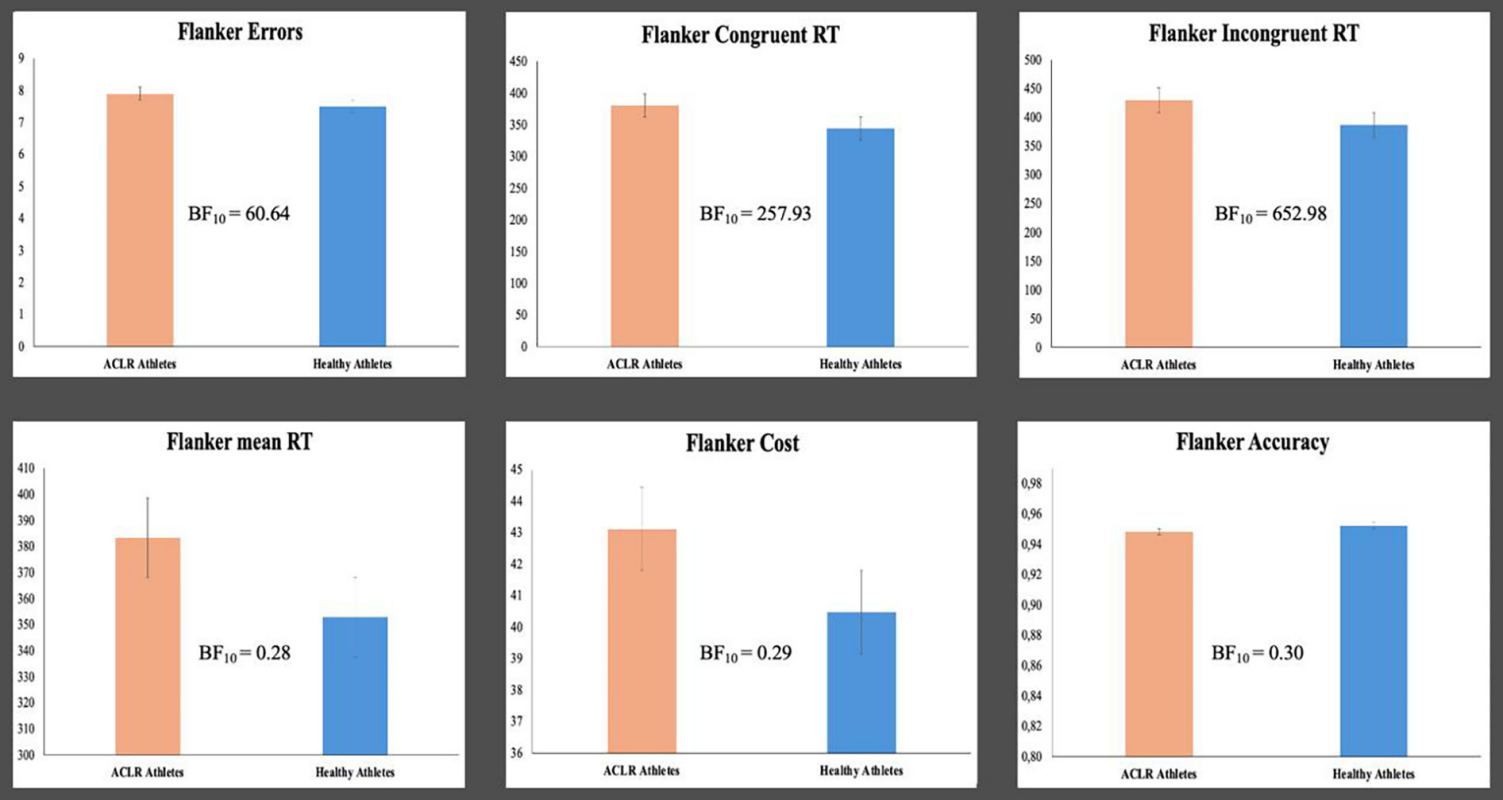
Differences in the flanker task between athletes with no history of ACL injury and athletes in the follow-up phase.

For the Flanker task, the results regarding means (SD) and Bayes factors BF_10_ are reported in Table 4 and Figure 2. Additionally, results for the Bayes factor BF_10_ are reported in Figure 2. There was substantial evidence in favor of the alternative hypothesis for mean overall reaction time, congruent reaction time, and incongruent reaction time, indicating that performance on these variables was superior for the healthy athlete group. In the case of errors, accuracy, and switching cost, substantial evidence was observed in favor of the null hypothesis, indicating that both groups performed similarly on these variables.

**Table 4:** Comparison of flanker task performance between athletes with no history of ACL injury and athletes in the follow-up phase.

Flanker task variables	Healthy athletes			ACLR athletes (follow-up)			
	M (SD)	95% credible interval		M (SD)	95% credible interval		BF_10_
		Lower	Upper		Lower	Upper	
Mean RT	352.85 (25.14)	343.46	362.24	385.17 (43.39)	357.60	412.73	44.28
Congruent RT	344.09 (24.14)	335.07	353.11	378.14 (41.04)	352.06	404.21	217.35
Incongruent RT	387.01 (35.88)	387.01	35.88	443.08 (32.77)	422.26	463.91	457.76
Errors	7.50 (3.99)	7.5	3.99	7.67 (5.43)	4.22	11.12	0.27
Mean accuracy	0.95 (0.03)	0.95	0.03	0.95 (0.03)	4.215	11.118	0.28
Cost of change	40.49 (15.74)	96.67	5.47	48.31 (8.46)	42.931	53.679	0.33

Data values are expressed as mean (standard deviation); ACLR, anterior cruciate ligament reconstruction; BF_10_, Bayes factor.

**Figure 3:**
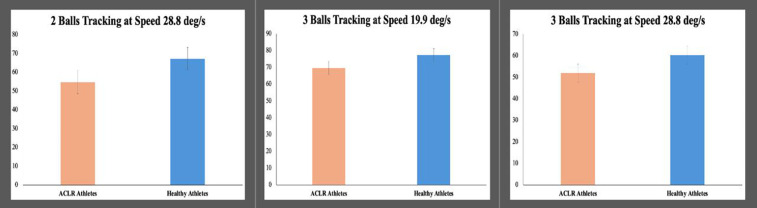
Differences in multiple object tracking between athletes with no history of ACL injury and athletes in the follow-up phase.

For the MOT test, the results regarding means (SD) and Bayes factors BF_10_ are reported in Table 5 and Figure 3. In the follow-up measure for the 2-ball condition at a speed of 28.8 deg/s and for the 3-ball condition at speeds of 19.9 and 28.8 deg/s, cognitive performance is superior for healthy athletes compared to injured athletes.

**Table 5:** Comparison of cognitive performance in the MOT task between athletes without a history of ACL injury and athletes in the follow-up phase.

MOT task variables	Healthy athletes			ACLR athletes (follow-up)			
	M (SD)	95% credible interval		M (SD)	95% credible interval		BF_10_
		Lower	Upper		Lower	Upper	
2 balls (6.4 deg/s)	96.67 (5.47)	94.63	98.71	95.83 (6.69)	91.59	100.08	0.27
2 balls (9.3 deg/s)	97.00 (5.35)	95.00	98.99	96.67 (4.92)	93.54	99.80	0.27
2 balls (13.7 deg/s)	90.67 (7.85)	87.74	93.60	89.33 (13.11)	75.72	97.62	0.29
2 balls (19.9 deg/s)	80.67 (10.48)	76.75	84.58	74.17 (10.84)	67.28	81.05	1.53
2 balls (28.8 deg/s)	67.00 (13.68)	61.89	72.11	53.33 (17.75)	42.05	64.61	18.84
3 balls (6.4 deg/s)	98.89 (2.54)	97.94	99.83	98.88 (2.61)	97.23	100.54	0.29
3 balls (9.3 deg/s)	96.54 (6.16)	93.24	97.84	97.56 (6.00)	93.40	101.03	0.39
3 balls (13.7 deg/s)	88.19 (7.77)	85.29	91.10	86.33 (9.15)	82.51	94.15	0.32
3 balls (19.9 deg/s)	77.31 (9.04)	73.93	80.68	72.50 (11.55)	65.16	79.84	16.30
3 balls (28.8 deg/s)	60.32 (10.88)	56.26	64.38	51.94 (3.42)	44.41	59.47	12.12

Data values are expressed as mean (standard deviation). ACLR, anterior cruciate ligament reconstruction; deg, degrees; s, seconds; BF_10_, Bayes factor.

It should be noted that after performing the analyses using alternative values of 0.2 and 1 for the prior, the results were not affected, ensuring their robustness. For example, for the Flanker task in the average general reaction time, BF_10_ = 40.82 was obtained with prior *r* = 0.05, BF_10_ = 23.82 with prior *r* = 0.02, and BF_10_ = 43.80 with prior *r* = 1. In the case of the MOT task for the speed of 3 balls at 19.9 deg/s, BF_10_ = 10.18 was obtained with prior *r* = 0.05, BF_10_ = 15.68 with prior *r* = 0.02, and BF_10_ = 15.49 with prior *r* = 1. In both tasks, these results indicate evidence favoring the alternative hypothesis.

## 4 Discussion

The main objective of this study was to understand how cognitive performance evolves between stages of the ACL injury recovery process: preoperative, postoperative, and at the end of the rehabilitation process. It was observed that athletes had worse cognitive performance in the preoperative phase compared to the postoperative and final phases of rehabilitation in the Flanker task and MOT. In particular, longer reaction times were observed on Flanker and worse tracking of three balls at speeds of 13.7, 19.9, and 28.8 deg/s. However, no effect was found between the postoperative and final phases of rehabilitation for any cognitive test. These data suggest that cognitive performance improved at the beginning of the rehabilitation phase after surgery, but this performance remained stable. Finally, it is important to note that, at the end of the recovery process, the rehabilitated athletes did not reach a level of cognitive performance comparable to that of those without a history of ACL injury.

Athletes performed worse during the MOT task preoperative phase than in later recovery phases. They also presented longer response times in the Flanker task, which shows greater difficulty in processing and resolving conflicting visual information at this stage. Given that the aforementioned variables directly influence the athlete's ability to process information and coordinate movements in uncertain environments (11), assessing these cognitive abilities, which do not appear to evolve during the recovery process, is presented as a key strategy to monitor the recovery process from a cognitive perspective. Although an improvement in cognitive performance was observed from the preoperative to the postoperative phase, no further progress was detected between the postoperative and follow-up phase, suggesting a possible plateau in recovery. This stabilization could reflect a true ceiling in functional recovery. However, alternative explanations must also be considered, such as ceiling effects in task sensitivity, the specificity of the cognitive domains assessed, or inter-individual variability in adaptation rates. The interpretation that this lack of change may be related to limitations in cognitive rehabilitation strategies or to pre-existing differences in baseline cognitive abilities should be viewed with caution and warrant further investigation. In this sense, it has been observed that athletes with poorer cognitive performance have a higher risk of musculoskeletal injury (16). Recently injured ACL athletes presented limited interference control capacity, likely aggravated by typical symptoms of the initial post-injury phase, such as reduced knee range of motion, proprioceptive impairment, and decreased joint stability (26). The beginning of the rehabilitation phase after surgery appears to allow the patient to improve these variables. This trend may be linked to the neuromuscular stimulation offered in this phase (34). On the other hand, the lack of improvement observed at the end of the rehabilitation process compared to the postoperative phase could indicate a lack of effectiveness of the strategies implemented to stimulate improved cognitive control in patients. Authors such as Criss et al. (35) highlight the need to include the cognitive demands of the competitive context where the injury occurs in rehabilitation programs to recover the adaptive capacity to this environment. Gokeler et al. (36) highlight the need to do so in injury prevention programs.

The group of ACL-injured athletes who had completed rehabilitation demonstrated poorer cognitive performance than their peers without a history of ACL injury. Behavioral performance on the interference control task and the MOT failed to reach the levels seen in athletes without a history of ACL injury. However, it is not possible to determine whether these differences are a direct consequence of the injury or whether they were already present before the injury and may have constituted a risk factor for the injury (17). One possible explanation for the lack of effect between the postoperative and final recovery phases is that after overcoming the acute phase following surgery, the athletes regained their initial cognitive level, which was already lower than that of athletes without a previous injury. This hypothesis suggests that the observed differences may not be due to poor rehabilitation but to pre-existing characteristics in these athletes. In this sense, the fact that athletes without a history of injury demonstrate better management of attentional resources and more efficient organization of their neural networks may be due to a more fluid interaction between the peripheral nervous system, which captures and transmits sensory information, and the central nervous system, responsible for processing and responding to it (37). On the other hand, the anterior cingulate cortex plays a crucial role in these processes by filtering distractions and prioritizing relevant stimuli, allowing for better conflict resolution (38). Conversely, an increase in response time could indicate a less efficient connection between these systems, affecting performance in demanding cognitive tasks (39).

The findings of this study highlight the critical role of executive functions in athletes recovering from ACL injury. This study evaluated two key domains of cognitive function, specifically cognitive inhibition (interference control) and dynamic attention. On the one hand, cognitive inhibition is the ability to suppress irrelevant or interfering information in order to respond correctly to target stimuli (40). This skill is vital for athletes, who must often ignore distracting cues (such as opponents' feints or crowd noise) and remain focused on task-relevant information during the competition (41). On the other hand, dynamic attention reflects the capacity to distribute and sustain attention on multiple moving objects simultaneously. In sports contexts, dynamic attention enables an athlete to track teammates, opponents, and the ball simultaneously, thereby anticipating play developments in real time (42). These executive functions are highly relevant for athletes recovering from an ACL injury. According to previous research, athletes rehabilitated from an ACL injury exhibit deficits in inhibitory control compared to athletes without a history of ACL injury (43). This might lead to slower or incorrect decision-making, for example, failing to filter out misleading movements by an opponent (17); while deficits in dynamic attentional tracking could make an athlete less aware of emerging threats or opportunities on the field (44). Such lapses in cognitive function during high-pressure, unpredictable game situations could increase the likelihood of re-injury or performance errors. Strengthening cognitive inhibition and dynamic attention alongside ACL recovery, rehabilitation programs can better prepare athletes for safe return-to-play, ensuring they are not only physically recovered but also cognitively prepared and capable to face the complex cognitive demands of their sport.

This study provides preliminary information on how cognitive performance evolves at different stages of ACL injury recovery. This study aims to improve interventions to restore cognitive abilities and prepare athletes to meet the specific demands of competition. However, this study has certain limitations that should be taken into account. On the one hand, the cognitive tests used in this study are general and are not tailored to the specific performance of the participants. Future research could assess cognitive performance through tasks that challenge athletes' cognitive resources in a specific and individualized manner, adjusting the cognitive demands to their particular abilities. Furthermore, due to the design of this study, it is important to highlight the impossibility of establishing a cause-effect relationship between cognitive deficits and the occurrence of ACL injury. A pre-injury assessment would determine whether the cognitive deficit is a direct consequence of the ACL injury or if it is one of the factors that predispose to injury. Hence, it would be necessary to monitor the cognitive performance of players in sports clubs throughout the season.

## 5 Practical applications

Our results support the importance of assessing athletes to identify potential cognitive deficits that could lead to a high risk of injury. Thus, this assessment process would become one of the strategies aimed at injury prevention.

Although a causal relationship cannot be established, the association found between ACL injury and cognitive deficit suggests the need to consider including greater cognitive stimulation in the ACL injury recovery process. From this perspective, ACL rehabilitation can induce cortical reorganization, changing how the brain processes motor and sensory information (45). To promote these changes, patients should be stimulated from the initial stages of rehabilitation, even when physical movement is restricted due to pain, impaired motor control, or joint immobilization. In these cases, it is essential to implement techniques such as motor imagery (the mental simulation of a specific movement without physical execution) and action observation (watching others perform goal-directed motor actions, often through video demonstrations), which have been proven effective in promoting adaptive neuroplasticity (46). In the context of ACL rehabilitation, observing or imagining sport-specific movements such as landings, changes of direction, or decelerations may enhance motor reprogramming and facilitate the transfer of cognitive-motor improvements to real performance settings. Along the same lines, Schnittjer et al. (47) point out that ACL injury compromises the mechanical structure of the knee and its neurosensory function, affecting the brain's ability to process information and coordinate movements. Therefore, they recommend incorporating rehabilitation approaches that reduce sensory uncertainty, such as training with enhanced feedback or using technologies such as virtual reality in dynamic stability tests and tasks with unexpected perturbations to measure responsiveness and optimize recovery.

In later stages, once the athlete has fully recovered, that is, has completed the rehabilitation phase under the supervision of a physiotherapist, the athlete should be referred to a physical exercise professional and/or sports rehabilitation specialist who will prescribe sports activities that combine physical and cognitive tasks simultaneously (e.g., performing balance exercises while responding to visual or auditory stimuli). In this regard (48), propose a model based on manipulating the functional task environment, incorporating uncertain and cognitively demanding situations. Therefore, incorporating exercises that manipulate cognitive demands during motor tasks typical of interactive sports, such as landings, decelerations, or changes of direction, may represent a promising strategy to enhance rehabilitation outcomes and potentially contribute to reducing re-injury risk, although further research is needed to confirm its effectiveness. To achieve this, cognitive demands can be increased by introducing uncertainty or using the dual-task paradigm (49). All of this could ensure the restoration of the mechanisms responsible for communication between the peripheral and central systems responsible for controlling and adjusting actions in uncertain contexts. In this way, athletes could develop physical and cognitive performance that allows them to adapt to complex and demanding situations, thereby reducing the risk of relapse.

## 6 Conclusion

Athletes who have suffered an ACL injury show improvements in cognitive performance during the postoperative phase and at the end of rehabilitation, compared to their performance during the preoperative phase. However, during rehabilitation, their cognitive performance stabilizes and remains lower than that of athletes who did not suffer the injury. This data suggests that ACL rehabilitation programs may not be sufficient to fully restore athletes' cognitive performance fully. Therefore, it is essential that, after the rehabilitation period developed by the physiotherapist, a sports and exercise professional complete the process with a physical readaptation phase that stimulates cognitive performance to recover specific functionality that will ensure adaptation to environmental stimuli in the uncertain environments typical of competition, reducing the risk of relapse.

## Data Availability

The raw data supporting the conclusions of this article will be made available by the authors, without undue reservation.
